# On the History of Dentistry from the Earliest Times to the Founding of the Profession by Hayden and Harris; with Special Reference to the Immediate Publication of Such a Work in English

**Published:** 1905-12

**Authors:** Charles M’Manus

**Affiliations:** Hartford, Conn.


### ON THE HISTORY OF DENTISTRY FROM THE EARLIEST TIMES TO THE FOUNDING OF THE PROFESSION BY HAYDEN AND HARRIS; WITH SPECIAL REFERENCE TO THE IMMEDIATE PUBLICATION OF SUCH A WORK IN ENGLISH.[1]

DR. CHARLES M'MANUS, HARTFORD, CONN.

WHEN I promised nearly a year ago to read a paper before this Institute, I had but a very vague idea of what I could present that would be of any particular interest to the members. That it should be something of a historical character goes without saying,—for I deal in little else. That it would probably be an appeal to you to take a little interest in the early development of that art which you are all engaged in developing was very probable.

But that I should be able to come before you this evening with the surprisingly good news that the *dental history* (for which we have looked so long,—with a sort of despair,—) was an accomplished fact, was more than I could have dreamed of, when I promised to appear before you.

The work exists,—within the past week I have read it with the deepest interest and admiration. It has been written, as I have always believed it should be written, by a dentist, and what is more remarkable by a dentist with the historical instinct,—a rather rare combination.

I refer to that distinguished dental historian Dr. Vincenzo Guerini, of Naples, Italy, and his "History of Dentistry from the most ancient times to the close of the Eighteenth Century."

The period I shall allude to in my remarks is the time from the very beginnings in medicine, dentistry, civilization itself, up to the true founding of the profession in this country by Hayden and Harris. I tell of dentistry before there was a society, a journal or a college, before anæsthesia: a dentistry without cohesive gold, the rubber dam, the engine, a true cement or amalgam and with porcelain only in a sort of crude infancy.

I propose towards the close of my remarks, this evening, to tell you of this work, but before doing so I must ask your kind

---

[1] Read before the New York Institute of Stomatology, October 3, 1905.

indulgence,—perhaps endurance,—while I briefly sketch the development of the dental art during the period covered,—in a most complete, in fact, exhaustive manner, by Dr. Guerini.

The first beginnings[2] of the dental art were undoubtedly the same as those of general medicine for it is evident that in primitive times, when the healing art was still in a rudimentary stage, no divisions can have existed in it. Amongst the peoples of ancient times the Egyptian nation was without doubt the one in which civilization first took its rise and had its earliest development. The Ebers papyrus, in the library of the University of Leipsic, the oldest medical manuscript known, treats of diseases of the teeth and gums. In this very interesting book of ancient Egypt, reaching as far back as 3700 years before Christ, no less than fourteen prescriptions for the treatment of dental diseases are found. At this very remote epoch dental medicine was a part of general medicine; we say dental *medicine* because the papyrus of Ebers does not mention dental surgery.

The initiator of this lost science was undoubtedly the Greek Æsculapius who flourished thirteen centuries before the Christian era and who on account of his healing abilities was raised to the rank of a god by the gratitude of mankind. [Space will not permit[3] of the publication, in full, of the paper which was illustrated with sixty lantern slides of portraits, instruments and appliances, etc. A brief résumé will show the scope of the lecture]

After speaking of the Greeks, with a passing allusion to the lack of any dental reference in Homer, Herodotus was quoted regarding the state of medicine in Egypt in his time; when, among many other "specialists," there were those in charge of the *disorders of the teeth.* What degree of development dentistry reached among the Egyptians unfortunately, as yet, we do not know. All the assertions regarding artificial teeth, pivot teeth and particularly teeth with gold fillings that are said to have been found in Egyptian mummies are, *as yet,* entirely devoid of foundation. And yet in spite of this, it is not admissible that the Egyptians should not have known of prosthetic dentistry when their neighbors the people of Phœnicia, a country with which they had very intimate commercial relations, practiced it. Lantern

---

[2] Guerini.

[3] Note by C. M.

slides were shown of photographs of prosthetic pieces found in the Phœnician necropolis at Sidon, dating as far back as four hundred years before Christ; as well as various Etruscan pieces of even earlier date. These evidences of early dental work are authentic and to be found in the Civic Museum and the collection of Count Bruschi at Corneto, Italy. An even more interesting prosthetic piece found in an Etruscan grave at Orvieto and now in the museum of the University of Ghent, was shown, as well as a piece of Etruscan dental work from a tomb at Valsiarosa now in the museum of Pope Julius at Rome. All these ancient appliances were shown in *fac simile* at the International Dental Congress at St. Louis in the archæological collection of Dr. Guerini, who received a gold medal.

The dental art among the *Romans* was referred to with illustrations of various instruments. During the many centuries that have been called the "middle ages" dentistry was in a state of marked decline; we know very little about it and the little that was achieved must be attributed to the *Arabs*. Rhazes, the initiator of the operation of filling teeth, using for this purpose a mixture of mastic and alum, was spoken of and *Abul-casis* was quoted at some length and several illustrations of the instruments and appliances of the time were shown upon the screen. Abul-casis in the XI century was the first author who took into serious consideration dental tartar and recommended that a scrupulous cleansing of the teeth should be performed. The chapter[4] relating to this "On the Scraping of the Teeth" is worthy of attention:

"Sometimes on the surface of the teeth, both inside and outside, as well as under the gums are deposited rough scales, of ugly appearance and black, green or yellow in color. This corruption is communicated to the gums and so the teeth are in process of time denuded. It is necessary for thee to lay the patient's head upon thy lap, and scrape the teeth and molars on which are observed either true incrustations or something similar to sand and this until nothing more remains of such substances and until also the dirty color of the teeth disappears, be it black or green or yellowish or of any other color. If a first scraping is sufficient, so much the better, if not, thou *shalt repeat it on the following day or even on the third or fourth day* until the desired purpose is obtained. Thou must know however that the teeth need scrapers

---

[4] Liber I. cap 29, Guerini.

of various shapes and figures, on account of the very nature of this operation. In fact the scalpel with which the teeth must be scraped on the inside is unlike that with which thou shalt scrape the outside and that with which thou shalt scrape the interstices between the teeth shall likewise have another shape. Therefore thou must have all this series of scalpels ready if it so pleases God." Abul-casis shows in his work fourteen figures of these scalers, which were shown on the screen.

Passing on to the Fifteenth Century attention was called to *Giovanni d'Arcoli,* known under the Latinized form of Arculanus, who was the first to mention the filling of teeth with gold. In speaking of the stopping of decayed teeth, he says that it must not always be carried out with the same substance but that in making choice of the material one should have regard to the temperament of the individual and to the condition of the gums and of the tooth. He counsels filling the teeth, in certain cases, with *gold leaf.* The use of gold in dental stoppings, therefore, dates back at least to A. D. 1450. Many other writers were mentioned, particularly the great *Eustachius* and a brief account was given of *Ambroise Paré,* the "foster father of dental surgery," with several portraits and illustrations of dental instruments from his works.

A very rare and curious portrait of *Nathaniel Highmore* was shown, as well as one of the father of scientific microscopy *van Leuwenhoek,* also instruments from the work of *Pierre Dionis* (1718). After a brief glance at some of the writers of the Seventeenth Century, the "father of dental surgery," the illustrious *Pierre Fauchard* was spoken of at some length. His portrait from the original now in possession of Dr. Viau, of Paris, was shown, as well as the title page and frontispiece of his work (1728) on dental surgery with a number of the plates illustrating extracting instruments of several kinds, scalers, pluggers, and various artificial dentures.

An account of Fauchard's great work "The Surgeon-Dentist" was given with much detail and the quotation of many important passages. Two are of particular interest. In the preface to his book Fauchard says: "There does not exist any public or private course of surgery in which the theory of dental maladies is amply taught and in which one can receive fundamental instruction in this art, so necessary for the healing of these maladies and of those of the neighboring parts. This branch of the art having been but little cultivated, if not wholly abandoned, by the most celebrated surgeons their negligence has caused it to fall into the hands of persons without theory and without experience, who practice it in a haphazard fashion guided neither by principles nor methods. In Paris it is only since 1700 that people's eyes have become opened to this abuse. In this town those who intend to become dentists are now obliged to undergo an examination, but although the examiners be most learned and well versed in all the other parts of surgery, I think, if I may be allowed to express an opinion, that as they do not ordinarily themselves practice dental surgery, it would not be amiss on these occasions to admit an able and experienced dentist, who might sound the aspirants as to the difficulties which have come before him in the course of the long practice of his art and who could communicate to them the means of surmounting them. In this way one would not have to admit that the knowledge of the greater part of "dental experts" is below "mediocrity."

That this high-minded professional man was among the first to realize the necessity for a higher dental education may be seen in the following quotation:

"It is strange," writes Fauchard, "that the sovereigns of foreign countries, the heads of republics and also the administrators of our own provinces, do not provide for the expense of sending young surgeons to Paris to be instructed in a part of surgery so essential, and notwithstanding, so ignored and neglected everywhere excepting in this great city, where it has reached its highest perfection both as regards the embellishment of the mouth and the cure of diseases, often of a most serious character. These scholars would, thereafter, form others and would render great services to their nation and their fellow citizens."[5]

A mechanical device, probably the "germ" of the dental engine, described by Fauchard and illustrated by *Jourdain* in 1756, a drawing of which was shown on the screen. A brief account of *John Hunter*, with portrait, was given, as well as of other early English dentists.

The early history of dentistry in America with many por-

---

[5] 2nd Ed. Vol. II, page 366.

traits, advertisements, etc., was considered, some of the men spoken of being Joseph Lemaire, James Gardette, Josiah Flagg, John Greenwood, Edward Hudson, John Randall, Leonard Koecker, John R. and Shearjashub Spooner, of all of whom portraits were shown as well as of a number of other prominent practitioners of the early part of the Nineteenth Century, Du Bois de Chemant,, Audibran, Serre, C. Starr Brewster, B. A. Rodrigues, Parmly, Solyman Brown, Merritt and others, ending with a brief account of the great labors of *Hayden* and *Harris.*

---

For the past ten years Dr. Guerini has been compiling a history of dentistry fom the earliest records down to the Nineteenth Century. As he says in the introduction to his work: "The end I proposed to myself was to write a history of dentistry that should be more complete, more circumstantial and more exact than those published hitherto and which instead of being, as are many of these works, simply a compilation, should represent in part, the fruits of personal research and scrupulous examination of a vast number of works of various kinds containing elements utilizable for a history of dental art and science."

Those who have been accustomed to regard dentistry as a modern art can have but little idea of the extent to which we are indebted to those of the middle ages and earlier for much that is woven into the fabric of our modern practice. The collecting and harmonizing of the data for this early historical record has been a labor of many years by this expert specialist. Dr. Guerini says: "I have carefully collected the greatest possible number of historical data, keeping in view the consideration that some facts, although of little value in themselves may acquire a certain referential importance for the student desirous of procuring historical notes relating to some particular point in dental science."

This work is now ready for publication in the English language. It has been the desire of its distinguished author that it shall make its first appearance in that language and under American auspices.

In a report made by the "Committee on Dental History" to the National Dental Association at the meeting held last summer at Buffalo it was stated that they were of the opinion that the National Dental Association should secure the honor of publishing this history by undertaking to guarantee the sale of a sufficient number of copies to practically cover the cost. The committee found that it would be necessary to fix the retail price of the work *fully illustrated* and bound in cloth, at $5, and that in order to guarantee the cost of an edition of 1000 copies it would require 700 subscriptions at $5 each to insure a fund of $3,500 to pay the cost of publication. It was suggested by the committee that the National Dental Association assume publication of this important work with the understanding that it shall not be published until not less than 700 subscriptions be received.

The association very willingly lent its moral support to the enterprise and the matter will soon be placed not only before the members of the dental profession in this country but of the entire world.

